# “Awake but elsewhere”: impact of augmented reality glasses on conscious sedation in balloon-in-basket pulsed field ablation

**DOI:** 10.1007/s10840-026-02372-0

**Published:** 2026-06-20

**Authors:** Roland Richard Tilz, Jan-Per Wenzel, Charlotte Eitel, Sorin Popescu, Mirco Küchler, Roman Mamaev, Zgjim Avdimetaj, Kohei Ukita, Karl-Heinz Kuck, Sascha Hatahet

**Affiliations:** 1 Department of Rhythmology, University Heart Center Lübeck, University Hospital Schleswig-Holstein, Campus, Ratzeburger Allee 160, 23562 Lübeck, Germany; 2 German Center for Cardiovascular Research (DZHK), Partner Site Hamburg/Kiel/Lübeck, Lübeck, Germany

**Keywords:** Pulmonary vein isolation, Pulsed field ablation, Conscious sedation, Atrial fibrillation, Patient-reported outcomes, Balloon-in-basket catheter

## Abstract

**Introduction:**

Balloon-in-basket pulsed field ablation (BiB-PFA) enables atrial fibrillation (AF) ablation under conscious sedation (CS). While augmented reality (AR) has been proposed to improve patient comfort during invasive procedures, its benefit in an already well-tolerated awake ablation setting remains uncertain.

**Objective:**

To evaluate the impact of adjunctive AR glasses on sedation requirements and patient comfort during BiB-PFA performed under CS.

**Methods:**

In this prospective, single-center study, 50 patients undergoing AF ablation with BiB-PFA were treated under a standardized CS protocol. Patients were allocated to either ablation without AR (*n* = 25) or ablation with adjunctive AR glasses (*n* = 25). Sedation requirements and patient-reported outcomes were assessed during ablation, as well as at 1-hour and 1-day post-procedure.

**Results:**

Baseline characteristics were comparable. All procedures were completed without conversion to deep sedation. Fentanyl requirements were lower in AR-group (1.1 vs. 1.4 µg/kg; *p* = 0.015), while other sedatives were similar. Intraprocedural pain was moderate and comparable. AR enhanced perceived calming effect (10.0 vs. 8.5; *p* = 0.015). Satisfaction was high in both groups. Recommendation scores were high overall, with higher values without AR (90(90,100) vs. 90 (70,90); *p* = 0.023). Retrospective anxiety at day 1 remained low but was higher with AR (2.0(0.0,5.0) vs. 0.0 (0.0,0.0); *p* = 0.002). Pain decreased over time in both (*p* < 0.001).

**Conclusion:**

CS for BiB-PFA is feasible and well tolerated. Intraprocedural pain was low, while satisfaction and recommendation were high in both groups, with slightly higher values without AR support. AR use was associated with modest reduced opioid requirements, suggesting a potential role in selected patients. Slightly higher retrospectively reported anxiety at 1 day in AR-group, while overall low, warrants further investigation.

**Graphical abstract:**

**Figure:**
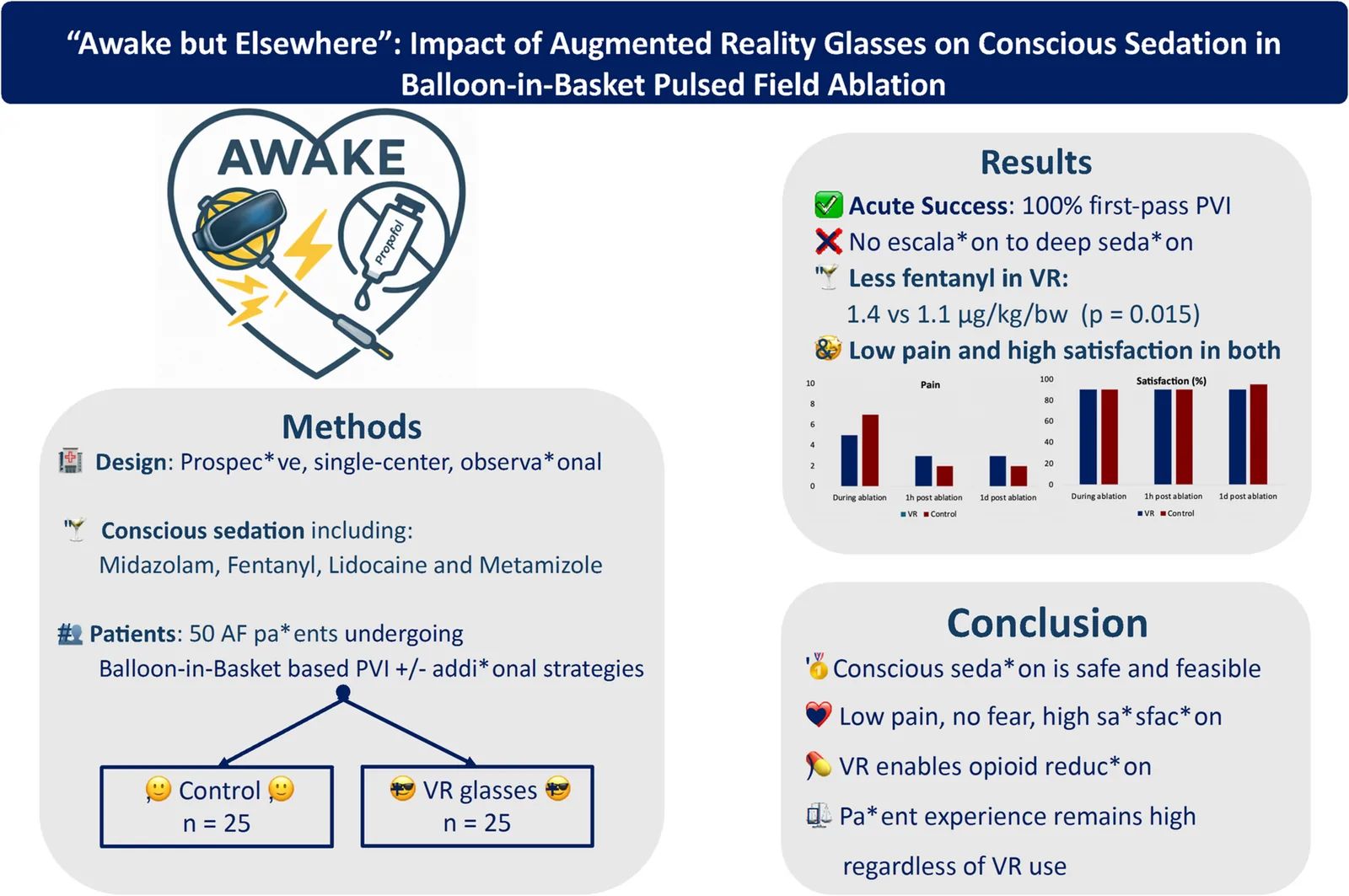

## Introduction

Pulsed field ablation (PFA) has become increasingly established for pulmonary vein isolation (PVI) in patients with atrial fibrillation (AF) [1]. The balloon-in-basket pulsed field ablation (BiB-PFA) catheter represents a novel system that combines circumferential ablation with integrated electroanatomical mapping. Its design allows stable positioning, homogeneous energy delivery, and simultaneous assessment of PVI, thereby integrating ablation and mapping within a single catheter platform.

Initial clinical experience with the BiB-PFA system has demonstrated that ablation can be performed safely and efficiently [2, 3]. In addition, early clinical experience suggests a favorable biomarker profile compared with other single-shot ablation technologies [4–7]. Beyond procedural efficacy, early observations suggest that this catheter design is associated with reduced extracardiac muscle contractions during energy delivery. This characteristic has enabled the performance of PVI under conscious sedation without propofol, avoiding the need for deep sedation or general anesthesia. In our initial experiences, patients remained communicative throughout the procedure and reported high levels of comfort and satisfaction [8].

While conscious sedation alone appears feasible in this setting, further optimization of patient comfort remains desirable, particularly with the aim of potentially lowering sedative and analgesic dosages. Evidence from interventional cardiology and other procedural disciplines suggests that immersive distraction techniques, especially augmented reality (AR) or virtual reality, may reduce anxiety, perceived pain, and sedative requirements during invasive procedures [9–11]. However, their benefit in the setting of an already well-tolerated awake ablation workflow remains unclear.

Based on these considerations, the present study was designed to evaluate the impact of adjunctive AR support on sedation requirements and patient-reported outcomes during BiB-PFA performed under conscious sedation.

## Methods

### Study population and trial design

This prospective, single-center study assessed the safety and feasibility of PVI performed with the BiB-PFA system under conscious sedation, comparing a standard awake protocol with an awake protocol supplemented by AR glasses, enabling real-time evaluation of procedural tolerance and patient experience.

Prior to the procedure, all patients received standardized information regarding the procedural steps and the planned sedation strategy. Patients with known anxiety or panic disorders were not included. All participants were informed that sedation could be escalated to deep sedation at any time during the procedure upon patient request.

Between May 2025 and March 2026, consecutive patients with symptomatic paroxysmal or persistent AF undergoing first-time PVI with or without additional atrial ablation strategies using the BiB-PFA catheter (VOLT™, Abbott) were enrolled. All procedures were performed under a conscious sedation protocol. Twenty-three patients underwent PVI under a standardized conscious sedation protocol without AR, followed by 25 consecutive patients treated with additional AR glasses (AR XREAL One Pro). Two further patients underwent ablation without AR to achieve balanced group sizes (*n* = 25 per group). Sixteen patients of the AR-group and one patient of the control group were enrolled in the VOLT™ CE extended study.

All participants were included in the Lübeck Ablation Registry. The study was approved by the local ethics committee and conducted in accordance with the Declaration of Helsinki [12]. Eligible patients were ≥ 18 years of age, had paroxysmal or persistent atrial fibrillation, and provided written informed consent.

### General procedural management

Detailed institutional BiB-PFA procedural workflows have been described previously [13, 14]. In summary, vascular access was obtained via two ultrasound-guided femoral venous punctures using 8 Fr sheaths after local anesthesia with 10 ml of 2% xylocaine. A diagnostic catheter was positioned in the coronary sinus. Transseptal access was achieved under fluoroscopic guidance using a modified Brockenbrough technique with an SL1 sheath (Abbott). Unfractionated heparin was administered immediately after transseptal puncture to maintain an activated clotting time > 300 s. The SL1 sheath was subsequently exchanged for a steerable 13 Fr sheath (Agilis™ NxT, Abbott), and pulmonary vein access was obtained over a 0.035″ guidewire.

To reduce vagal reactions, 1 mg atropine was administered intravenously prior to ablation. PFA was delivered at 1800 V with at least two rotated applications per vein (maximum of eight). Phrenic nerve capture was assessed by spline pacing during ablation of right-sided veins; if capture occurred, energy delivery was continued at 1400 V with a minimum of three applications. When posterior or anterior areas of fibrosis were identified by electroanatomic mapping using the BiB-PFA system, targeted ablation was performed with selective deactivation of non-contacting splines. The total number of applications and the choice between standard and reduced energy settings were left to the operator’s discretion.

Bidirectional conduction block was confirmed by demonstration of complete entrance and exit block across the respective lesion sets. Further procedural details regarding posterior wall ablation using the BiB-PFA system have been described in our recently published work [15]. In patients with concomitant typical atrial flutter, cavotricuspid isthmus ablation was performed with subsequent verification of bidirectional block using differential pacing. In patients undergoing superior vena cava (SVC) isolation, sinus rhythm was restored if necessary prior to ablation. An activation map of the right atrium and SVC was created using the BiB-PFA catheter to identify and avoid the sinus node region. After contrast delineation of the SVC–RA junction and assessment of phrenic nerve capture by high-output pacing, energy was delivered at 1800 V or reduced to 1400 V in case of phrenic capture. A single test application was performed to assess sinus node response before completion of circumferential SVC isolation with remapping to confirm electrical isolation, according to the previously published protocol [13]. Nitroglycerin was not administered at any time during the procedural workflow in this cohort. Post-ablation remapping was performed using the BiB-PFA system. In 17 patients, ablation followed the VOLT™ CE extended protocol. Phrenic nerve capture was first confirmed via a CS catheter. After CS placement, left atrial mapping was performed using the BiB-PFA-catheter. Following a 20-minute waiting period, remapping with the BiB-PFA-catheter and repeat phrenic nerve testing were performed to confirm durable isolation and phrenic integrity.

### Mild-sedation approach

All procedures followed a standardized institutional analgesia protocol tailored for awake ablation with the BiB-PFA system. Sedation was performed accordance with the current position paper issued by the national professional society [16].

The standardized multimodal analgesic protocol consisted of metamizole, fentanyl, midazolam, and intravenous lidocaine [8]. Lidocaine was included to support opioid-sparing analgesia and may additionally reduce coughing during energy delivery, thereby improving procedural stability and patient comfort [17–19]. Premedication began 15–30 min before the procedure with intravenous metamizole (1 g) and fentanyl (25 µg). Upon arrival in the EP laboratory, patients received an additional 1 g metamizole, 25 µg fentanyl, and 2 mg midazolam. Prior to procedure start, a dedicated team briefing was conducted, assigning one nurse exclusively to the patient’s head to ensure continuous communication, monitor comfort, and provide reassurance throughout the procedure.

In patients assigned to the AR-group, the AR glases was fitted immediately after arrival in the EP laboratory; patients were free to select their own film or series, while calming wildlife documentaries were displayed if no preference was expressed.

Fentanyl was titrated up to 1 µg/kg body weight prior to transseptal puncture. After completion of the second metamizole infusion, lidocaine (1.5 mg/kg) was administered slowly in 100 ml saline via peripheral venous access. Supplemental midazolam (1–2 mg) and fentanyl (25 µg) were administered as needed based on patient-reported discomfort. Propofol was reserved exclusively for direct-current cardioversion, which was performed only at the end of the procedure if required. Hemodynamics, oxygen saturation, ECG, and vigilance were continuously monitored. All medications were administered by trained EP nursing staff under electrophysiologist supervision. To preserve autonomy, participants were explicitly asked during the procedure whether they wished to convert to deep sedation. The protocol was designed to provide effective analgesia and anxiolysis while maintaining spontaneous respiration, full consciousness, and continuous communication—enabling real-time symptom monitoring and individualized titration.

### Patient-reported outcomes

Patient experience and procedural tolerability were systematically assessed using structured paper-based questionnaires at three predefined time points: during the procedure, 1 h post-ablation and on the day following the procedure. All assessments were conducted under staff supervision to ensure data accuracy.

During the procedure, patients were asked to rate pain intensity during PFA applications, dyspnea, and procedural anxiety using numeric rating scales from 0 to 10 [20]. These assessments were completed immediately after each ablation application to capture real-time perception of physical and emotional burden.

Immediately after the final ablation, patients rated their overall satisfaction with the sedation strategy (0–100%) and whether they would recommend it (0–100%).

At the same time point, patients were asked to reflect on their sensory experience during the procedure. Patients were asked whether they had perceived surrounding procedural activity—such as staff communication or ambient sounds—and, if so, to rate how calming or distracting these impressions were on a 0–10 scale. Physical autonomy was assessed by documenting whether patients were able to transfer independently from the procedure table to the hospital bed.

In patients assigned to the AR-group, additional questionnaire items specifically addressed the AR experience. These included ratings of headset comfort, perceived effects on anxiety reduction and pain relief, and occurrence of AR-related side effects such as cyber sickness, assessed using numeric rating scales ranging from 0 to 10. Overall evaluation of the AR experience and willingness to recommend awake PVI with AR support were assessed using percentage-based visual analog scales (0–100%), with higher values indicating greater satisfaction.

The same core questions were repeated 1 h and 1 day after ablation. At these time points, patients were asked to retrospectively evaluate the intensity of pain, dyspnea, and anxiety they had experienced during the ablation and whether they still felt any of these symptoms at the time of reassessment.

### Postprocedural management

**Fig. 1:**
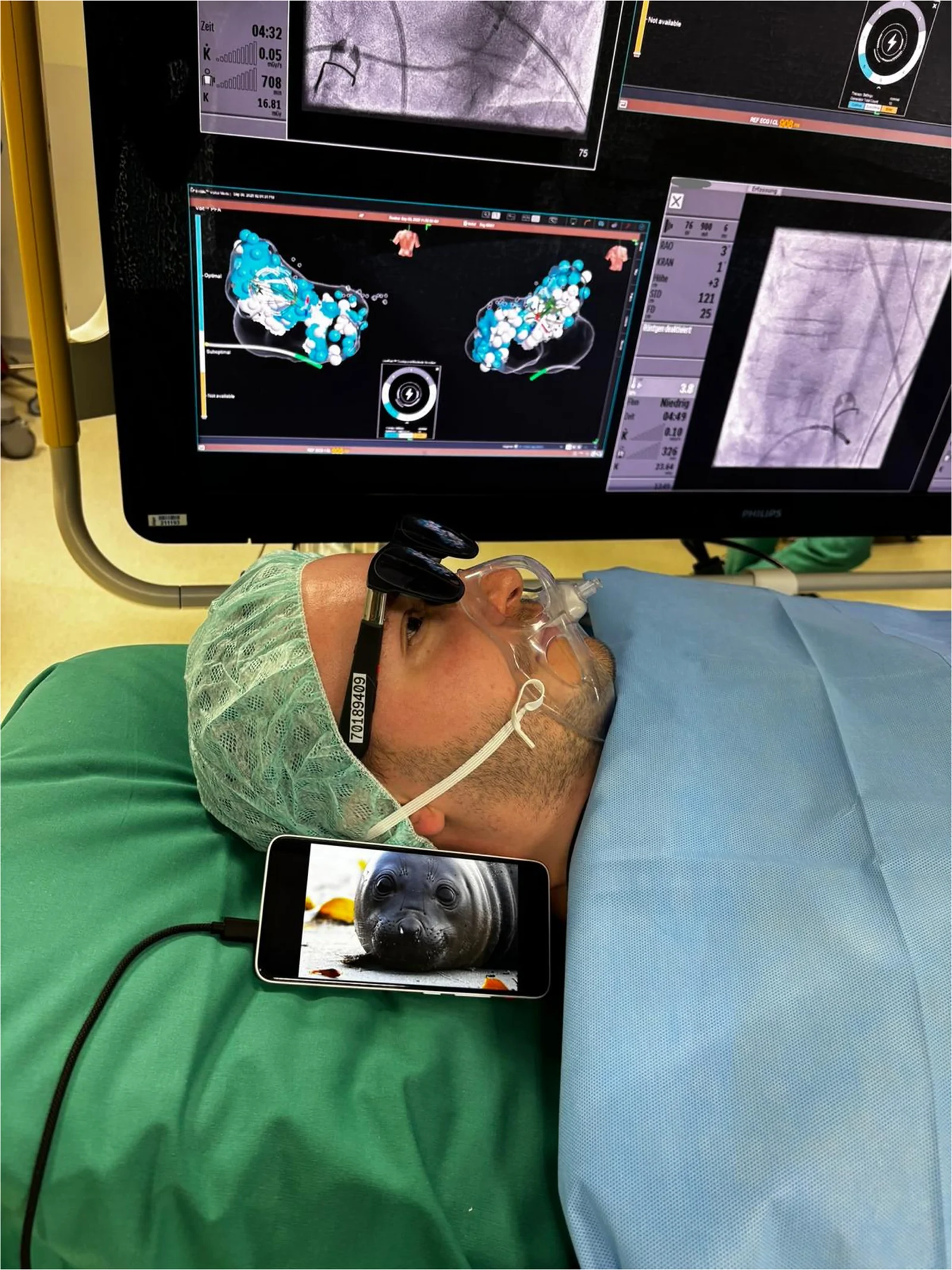
AR glasses. Representative image of AR glasses used during ablation while watching a wildlife documentary. (Written informed consent for image publication obtained)

**Fig. 2:**
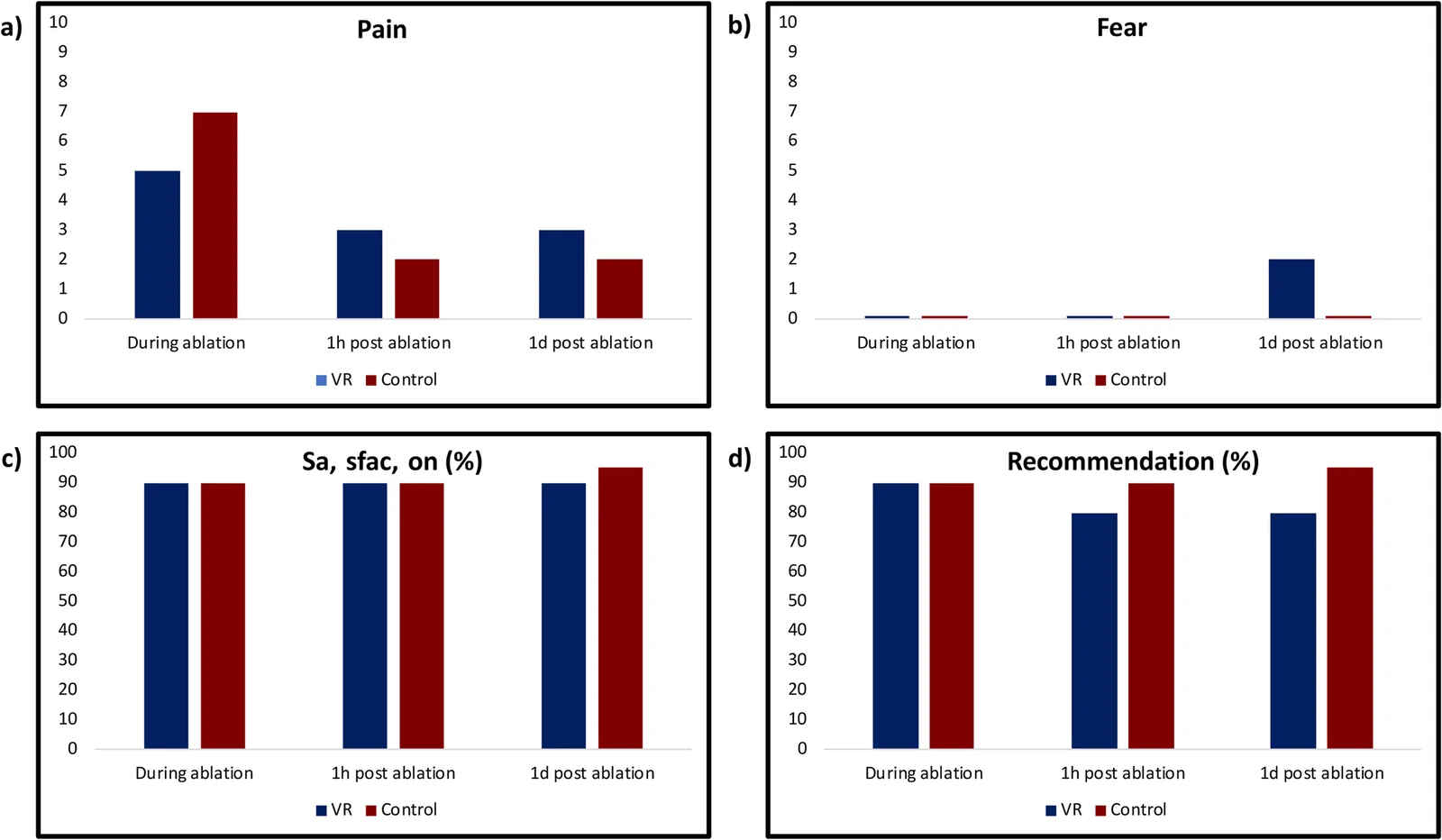
Changes in patient-reported parameters over time. Panels show (**a**) pain, (**b**) fear, (**c**) satisfaction, and (**d**) recommendation for AR and control groups during ablation, 1-hour, and 1-day post-procedure. Pain and fear are reported on a 0–10 scale, whereas satisfaction and recommendation are presented as percentages (0–100%)

Hemostasis was achieved using vascular closure devices or a figure-of-eight suture with compression. Pressure bandages were removed after 1 to 4 h, depending on the closure method. Sutures were taken out the next day. Transthoracic echocardiography was routinely performed immediately after the procedure, at one hour, and on the first post-procedural day to exclude pericardial effusion. Oral anticoagulation was resumed six hours after ablation and continued for at least two months. Long-term anticoagulation was guided by individual thromboembolic risk according to the CHA₂DS₂-VA score [21] (Figs. 1, 2).

### Statistical analysis

Continuous data were tested for normality using the Shapiro–Wilk test and are presented as median (Q1, Q3). Between-group comparisons were made using independent-samples t-tests or Mann–Whitney U tests. Categorical variables are reported as absolute and relative frequencies and compared using Fisher’s exact or Chi-square tests, depending on sample size. Temporal changes in patient-reported were analyzed using paired-samples t-tests or Wilcoxon signed-rank tests. Categorical variables are reported as counts and percentages. All analyses were performed using IBM SPSS Statistics 29.0.1.0 (IBM Corp., Armonk, NY, USA). All tests were two-sided; *p* < 0.05 was considered statistically significant.

## Results

### Patient and procedural characteristics

A total of 50 consecutive patients (32% female, age 68.0 (63.8, 75.0) years, paroxysmal AF 52%) underwent PVI using the BiB-PFA system under conscious sedation. Baseline characteristics were comparable between groups.

Procedural duration was longer in the AR-group (54.0 (46.0, 56.0) vs. 39.5 (34.8, 54.3) min; *p* = 0.030), as was left atrial dwell time (37.0 (25.0, 44.0) vs. 21.0 (19.8, 25.0) min; *p* = 0.005). PVI was achieved in all patients, with a comparable number of applications per vein. Posterior wall ablation was more frequent in the AR-group (84% vs. 28%; *p* < 0.001). In total, anterior wall ablation was performed in 4%, CTI ablation in 4%, and SVC isolation in 8% of patients. Electrical cardioversion rates were similar (40% vs. 36%; *p* = 0.771). No intraprocedural complications occurred in either group Table 1.

**Table 1:** Baseline characteristics and procedural metrics

Variable	Overall (*n* = 50)	Control (*n* = 25)	AR (*n* = 25)	*p*
Gender, female n (%)	16 (32)	7 (28%)	9 (36%)	0.747
Age	68.0 (63.8, 75.0)	68.0 (63.4, 75.0)	68.0 (63.0, 75.0)	0.942
BMI	27.7 (25.0, 29.4)	27.5 (24.9, 28.8)	28.0 (25.3, 29.7)	0.548
PAF, n (%)	26 (52)	10 (40%)	16 (64%)	0.265
CHA2DS2VASc-Score	2.0 (1.0, 3.0)	2.0 (1.0, 3.0)	2.0 (2.0, 3.0)	0.478
NTproBNP (ng/l)	462.0 (169.0, 997.0)	606.0 (139.0, 1013.0)	451.0 (229.0, 949.0)	0.947
Creatine (µmol/l)	77.0 (66.3, 90.3)	80.5 (72.7, 100.0)	71.0 (62.3, 87.7)	0.090
eGFR (mL/min)	83.0 (64.5, 91.0)	71.0 (64.0, 89.0)	86.0 (70.0, 97.0)	0.202
Procedure duration (min)	49.0 (37.0, 55.0)	39.5 (34.8, 54.3)	54.0 (46.0, 56.0)	0.030
Fluoroscopy Time (min)	6.5 (5.2, 9.0)	6.3 (4.6, 7.2)	7.3 (5.4, 10.0)	0.078
VOLT LA dwelling (min)	25.0 (20.0, 39.0)	21.0 (19.8, 25.0)	37.0 (25.0, 44.0)	0.005
CM (ml)	40.0 (30.0, 40.0)	40 (40, 50.0)	37.5 (30, 40)	0.168
LSPV applications	3.5 (2.0, 4.0)	3.0 (2.0, 4.0)	4.0 (3.0, 4.0)	0.192
LSPV, nominal energy n (%)	50 (100)	25 (100)	25 (100)	1.00
LIPV applications	3.5 (3.0, 4.0)	3.0 (2.0, 4.0)	4.0 (3.0, 4.0)	0.192
LIPV, nominal energy n (%)	50 (100)	25 (100)	25 (100)	1.00
RIPV applications	3.0 (2.8, 4.0)	3.0 (2.0, 4.0)	3.0 (3.0, 4.0)	0.296
RIPV, nominal energy n (%)	43 (86%)	21 (84)	22 (88)	0.416
RSPV applications	4.0 (2.0, 4.0)	4.0 (2.0, 4.0)	4.0 (3.0, 4.0)	0.765
RSPV, nominal energy n (%)	39 (78)	17 (68)	22 (88)	0.022
PW ablation	28 (56)	7 (28)	21 (84)	< 0.001
PW applications	4.0 (3.0, 5.0)	4.0 (4.0, 5.0)	4.0 (3.0, 5.0)	0.909
PW, nominal energy n (%)	28 (100)	17 (100)	22 (100)	1.000
AW ablation	2 (4)	2 (8)	0 (0)	0.490
AW applications	6.0 (5.5, 6.5)	6.0 (5.5, 6.5)	-	
AW, nominal energy n (%)	2 (100)	2 (100)	-	
CTI ablation	2 (4)	2 (8)	0 (0)	0.490
CTI applications	3.0 (2.0, 4.0)	3.0 (2.0, 4.0)	-	
CTI, nominal energy n (%)	2 (100)	2 (100)	-	
SVC isolation	7 (14)	4 (16)	3 (12)	0.609
SVC applications	2.5 (2.0, 3.5)	2.0 (2.0, 2.0)	3.0 (2.5, 4.0)	0.637
SVC, nominal energy n (%)	4 (100)	1 (100)	3 (100)	1.000
Electrical cardioversion	19 (38)	9 (36)	10 (40)	0.771

*AL* Anterior Linear Lesion, *BMI* Body Mass Index, *CTI* Cavo-Tricuspidal-Isthmus, *LA* Left Atrium, *LIPV* Left Inferior Pulmonary Vein, *LSPV* Left Superior Pulmonary Vein, *PAF* Paroxysmal Atrial Fibrillation, *PW* Posterior Wall, *RIPV *Right Inferior Pulmonary Vein, *RSPV* Right Superior Pulmonary Vein, *SVC* Superior Vena Cava

### Sedation and analgesia

No escalation to deep sedation was required in any patient. Overall, fentanyl was administered at a median dose of 1.2 (1.0, 1.5) µg/kg, midazolam 0.06 (0.04, 0.06) mg/kg, and lidocaine 1.5 (1.4, 1.5) mg/kg. Fentanyl requirements were significantly lower in the AR-group (1.1 (0.8, 1.3) vs. 1.4 (1.1, 1.6) µg/kg; *p* = 0.015), while midazolam (0.06 (0.05, 0.06) vs. 0.05 (0.04, 0.06) mg/kg; *p* = 0.938) and lidocaine (1.5 (1.4, 1.5) vs. 1.5 (1.4, 1.6) mg/kg; *p* = 0.587) were comparable between groups. Metamizole (2 g in all patients) and propofol use for cardioversion were similar. No AR-related side effects, including cyber sickness, nausea, or vomiting, were reported Table 2.

**Table 2:** Drug dosages per patient

Variable	Overall (*n* = 50)	Control (*n* = 25*)*	AR (*n* = 25)	*p*
Fentanyl (µg) – total Fentanyl µg/kg/ bw	100.0 (75.0, 125) 1.2 (1.0, 1.5)	125.0 (100.0, 125) 1.4 (1.1, 1.6)	100.0 (75.0, 112.5) 1.1 (0.8, 1.3)	0.015
Midazolam (mg) Midazolam mg/kg/ bw	5.0 (3.0, 5.0) 0.06 (0.04, 0.06)	4.0 (3.0, 6.0) 0.05 (0.04, 0.06)	5.0 (3.0, 5.0) 0.06 (0.05, 0.06)	0.938
Lidocaine (mg) Lidocaine mg/kg/ bw	128.8 (110, 147.5) 1.5 (1.4, 1.5)	127.5 (100, 140.0) 1.5 (1.4, 1.6)	130.0 (112.0, 150.0) 1.5 (1.4, 1.5)	0.587
Metamizole (mg)	2	2	2	
Propofol (mg) (Cardioversion only)	47.5 (30.0, 57.5)	55.0 (26.3, 100.0)	40.0 (30.0, 50.0)	

*bw* body weight

### Patient-reported outcomes

During energy delivery, patients reported moderate pain levels, which were numerically lower in the AR-group (5.0 (4.0, 7.0) vs. 7.0 (4.0, 8.0); *p* = 0.336). Dyspnea, groin pain, and procedural anxiety were low and comparable between groups.

The majority of patients (84%) reported awareness of surrounding procedural activity, which was generally perceived as calming, with significantly higher ratings in the AR-group (10.0 (8.0, 10.0) vs. 8.5 (6.0, 9.3); *p* = 0.015).

Immediately after the procedure, overall satisfaction was high in both groups (90 (83, 100)), without significant differences. Recommendation scores were similarly high, with slightly higher values observed in patients without AR support (90 (90, 100) vs. 90 (70, 90); *p* = 0.023). Physical autonomy after the procedure was comparable between groups.

At 1-hour post-procedure, retrospective ratings of intraprocedural pain were markedly reduced in both groups, without differences between AR and non-AR patients. Retrospective perception of dyspnea, anxiety, and groin discomfort during the procedure remained low and comparable between groups.

At 1-day follow-up, retrospective perception of intraprocedural pain remained low in both groups. Retrospective ratings of dyspnea and groin discomfort remained minimal and comparable. Anxiety levels were overall low, with slightly higher retrospectively reported values in the AR-group (2.0 (0.0, 5.0) vs. 0.0 (0.0, 0.0); *p* = 0.002). Overall satisfaction and recommendation remained high without significant differences.

Across all patients, retrospective pain perception decreased significantly over time compared to intraprocedural ratings (*p* < 0.001), while satisfaction and recommendation remained stable. Between-group comparisons of temporal changes showed no significant differences for pain, dyspnea, satisfaction, or recommendation, while a significant difference was observed for anxiety at 1-day follow-up (*p* = 0.037) Tables 3, 4, 5, 6, and 7.

**Table 3:** Patient questionnaire during ablation

Variable	Overall (*n* = 50)	Control (*n* = 25*)*	AR (*n* = 25)	*p*
Pain during application (0–10)	6.0 (4.0, 7.8)	7.0 (4.0, 8.0)	5.0 (4.0, 7.0)	0.336
Vein with highest pain	RSPV (32%)	RSPV (36%)	RSPV (28%)	
Groin pain during ablation (0–10)	0.0 (0.0, 2.0)	1.0 (0.0, 2.0)	0.0 (0.0, 2.0)	0.233
Fear during ablation (0–10)	0.0 (0.0, 1.0)	0.0 (0.0, 1.0)	0.0 (0.0, 1.0)	0.562
Dyspnea during ablation (0–10)	0.0 (0.0, 1.0)	0.0 (0.0, 1.0)	0.0 (0.0, 0.0)	0.061
Hearing surrounding?	42 (84%)	21 (84%)	21 (84%)	1.000
If yes, how calming? (0–10)	9.0 (8.0, 10.0)	8.5 (6.0, 9.3)	10.0 (8.0, 10.0)	0.015
Overall satisfaction (0-100%)	90 (83, 100)	90 (90, 100)	90 (80, 90)	0.111
Recommendation (0-100%)	90 (80, 100)	90 (90, 100)	90 (70, 90)	0.023
Moving from table by its own (0–10)	6.0 (4.0, 7.0)	5.0 (4.0, 6.6)	6.0 (4.8, 7.0)	0.445

**Table 4:** Patient questionnaire one hour following ablation

Variable	Overall (*n* = 50)	Control (*n* = 25*)*	AR (*n* = 25)	*p*
Pain during application (0–10)	2.5 (0.0, 6.8)	2.0 (0.0, 5.0)	3.0 (0.0, 7.0)	0.976
Pain now (0–10)	0.0 (0.0, 0.0)	0.0 (0.0, 0.0)	0.0 (0.0, 0.0)	0.162
Groin pain during ablation (0–10)	0.0 (0.0, 0.0)	0.0 (0.0, 0.0)	0.0 (0.0, 0.0)	0.550
Groin pain now (0–10)	0.0 (0.0, 1.0)	0.0 (0.0, 1.0)	0.0 (0.0, 1.0)	0.164
Fear during ablation (0–10)	0.0 (0.0, 1.0)	0.0 (0.0, 0.0)	0.0 (0.0, 3.0)	0.085
Fear now (0–10)	0.0 (0.0, 0.0)	0.0 (0.0, 0.0)	0.0 (0.0, 0.0)	0.556
Dyspnea during ablation (0–10)	0.0 (0.0, 0.0)	0.0 (0.0, 0.0)	0.0 (0.0, 0.0)	0.441
Dyspnea now (0–10)	0.0 (0.0, 0.0)	0.0 (0.0, 0.0)	0.0 (0.0, 0.0)	0.337
Hearing surrounding?	36 (72%)	16 (64%)	20 (80%)	0.175
If yes, how calming? (0–10)	8.0 (6.0, 10.0)	8.0 (6.0, 8.0)	10.0 (7.3, 10.0)	0.142
Overall satisfaction (0-100%)	90 (80, 100)	90 (90, 100)	90 (80, 100)	0.614
Recommendation (0-100%)	90 (80, 100)	90 (80, 100)	80 (80, 100)	0.313

**Table 5:** Patient questionnaire one day following ablation

Variable	Overall (*n* = 50)	Control (*n* = 25*)*	AR (*n* = 25)	*p*
Pain during application (0–10)	2.0 (0.0, 5.0)	2.0 (0.0, 4.3)	3.0 (0.0, 5.0)	0.762
Pain now (0–10)	0.0 (0.0, 0.0)	0.0 (0.0, 0.0)	0.0 (0.0, 0.0)	1.000
Groin pain during ablation (0–10)	0.0 (0.0, 1.0)	0.0 (0.0, 1.0)	0.0 (0.0, 0.0)	0.026
Groin pain now (0–10)	0.0 (0.0, 2.0)	0.0 (0.0, 2.0)	1.0 (0.0, 2.0)	0.157
Fear during ablation (0–10)	0.0 (0.0, 2.0)	0.0 (0.0, 0.0)	2.0 (0.0, 5.0)	0.002
Fear now (0–10)	0.0 (0.0, 0.0)	0.0 (0.0, 0.0)	0.0 (0.0, 0.0)	1.000
Dyspnea during ablation (0–10)	0.0 (0.0, 0.0)	0.0 (0.0, 0.0)	0.0 (0.0, 0.0)	0.258
Dyspnea now (0–10)	0.0 (0.0, 0.0)	0.0 (0.0, 0.0)	0.0 (0.0, 0.0)	0.332
Hearing surrounding?	32 (64%)	16 (64%)	16 (64%)	1.000
If yes, how calming? (0–10)	8.0 (6.0, 10.0)	8.0 (6.5, 9.0)	7.0 (5.0, 10.0)	0.662
Overall satisfaction (0-100%)	90 (80, 100)	95 (80, 100)	90 (80, 100)	0.199
Recommendation (0-100%)	90 (80, 100)	95 (80, 100)	80 (80, 100)	0.376

**Table 6:** Patient questionnaire: Comparison one hour versus ablation, one day versus ablation

Variable	Delta 1 h	*p*- value	Delta 1d	*p*- value
Overall				
Pain during application (0–10)	-2.0 (-5.0, 0.0)	< 0.001	-2.5 (-5.0, 0.3)	< 0.001
Fear during ablation (0–10)	0.0 (-0.8, 0.8)	0.140	0.0 (-1.0, 2.0)	0.028
Dyspnea during ablation (0–10)	0.0 (0.0, 0.0)	0.154	0.0 (0.0, 0.0)	0.167
Overall satisfaction (0-100%)	0.0 (0.0, 1.0)	0.724	0.0 (-1.0, 1.0)	0.403
Recommendation (0-100%)	0.0 (-0.8, 0.8)	0.575	0.0 (-1.8, 1.0)	0.383
AR				
Pain during application (0–10)	-2.0 (-4.0, 0.0)	0.006	-2.0 (-5.0, 0.0)	0.001
Fear during ablation (0–10)	0.0 (-1.0, 3.0)	0.063	2.0 (-0.1, 5.0)	0.007
Dyspnea during ablation (0–10)	0.0 (0.0, 0.0)	0.098	0.0 (0.0, 0.0)	0.098
Overall satisfaction (0-100%)	0.0 (0.0, 1.0)	0.581	0.0 (-1.0, 1.0)	0.266
Recommendation (0-100%)	0.0 (-1.0, 1.0)	0.575	0.0 (-2.0, 1.0)	0.910
Control				
Pain during application (0–10)	-2.0 (-4.0, 0.0)	0.003	-4.0 (-6.0, -1.0)	0.002
Fear during ablation (0–10)	0.0 (-1.0, 3.0)	0.608	0.0 (-1.0, 0.0)	0.437
Dyspnea during ablation (0–10)	0.0 (0.0, 0.0)	0.724	0.0 (-1.0, 0.0)	0.179
Overall satisfaction (0-100%)	0.0 (0.0, 1.0)	0.963	0.0 (-1.0, 1.0)	0.954
Recommendation (0-100%)	0.0 (-1.0, 3.0)	0.306	0.0 (-1.0, 1.0)	0.241

**Table 7:** Between-group comparison of changes in patient-reported outcomes at 1 h and 1 day

Variable	Delta 1 h *p*- value	Delta 1d *p*- value
Pain during application (0–10)	0.182	0.359
Fear during ablation (0–10)	0.242	0.037
Dyspnea during ablation (0–10)	0.990	0.364
Overall satisfaction (0-100%)	0.569	0.711
Recommendation (0-100%)	0.242	0.162

## Discussion

This prospective study is the first to systematically evaluate the impact of adjunctive AR support on sedation requirements and patient-reported outcomes during AF ablation using the BiB-PFA system under conscious sedation. The concept of “awake ablation” has gained increasing interest to reduce sedation-related risks and procedural complexity, yet data on strategies to further optimize patient comfort in this setting remain limited.

The present findings support the feasibility and safety of this minimalist sedation strategy. Key observations from the study include:

- Conscious sedation enabled successful completion of all procedures without the need for escalation to deep sedation.
- Intraprocedural pain and anxiety levels were low, with high overall satisfaction and recommendation rates.
- AR support was associated with a modest reduction in opioid requirements.
- Patient-reported experience remained highly favorable in both groups, with slightly higher satisfaction and recommendation in patients without AR support.
- Retrospectively reported anxiety at 1 day was slightly higher in the AR-group, while overall remaining low.

### Conscious sedation as a procedural strategy

In contemporary electrophysiology practice, PFA based PVI is predominantly performed under deep sedation or general anesthesia to ensure procedural control. However, this approach is associated with increased resource use and clinical risks, including respiratory compromise or hemodynamic instability. In addition, propofol use is restricted in many European healthcare systems, often requiring anesthesiology support and limiting procedural flexibility [22–24].

Against this background, conscious sedation has emerged as an attractive alternative, aiming to maintain spontaneous ventilation and patient interaction while providing adequate analgesia and comfort [8, 25, 26]. In the present study, this approach proved robust, with all procedures completed without escalation to deep sedation and consistently high patient satisfaction and recommendation. Beyond safety, conscious sedation offers practical advantages, including streamlined workflows, greater procedural flexibility, and reduced dependency on anesthesiology support.

### Patient comfort and procedural experience

Overall, patient-reported outcomes were highly favorable and in line with previous studies [8, 26]. Intraprocedural pain was moderate to low and decreased markedly on retrospective assessment, while dyspnea and anxiety remained minimal. Satisfaction and recommendation were consistently high, confirming excellent patient acceptance of the awake approach.

A key component of this workflow is continuous patient–staff interaction. Dedicated personnel providing communication, reassurance, and real-time response to patient feedback likely play a central role in perceived safety and comfort, representing an important non-pharmacological determinant of procedural tolerance.

### Augmented reality: potential benefits and limitations

The addition of AR support was associated with a modest but significant reduction in opioid requirements, suggesting a potential role as an adjunctive tool to optimize analgesia. In addition, patients in the AR-group reported a more calming perception of the procedural environment.

However, these effects did not translate into improved overall patient experience. The absence of further improvement in satisfaction may reflect a ceiling effect, as patient experience was already highly favorable under the standardized sedation protocol, potentially masking incremental benefits of AR. Although satisfaction and recommendation were high in both groups, with numerically slightly higher values in patients without AR support, these differences should be interpreted in the context of overall high baseline levels. One possible explanation is that while AR provides distraction from procedural stimuli, it may also attenuate the beneficial effects of direct communication between patient and staff. In an awake setting, continuous verbal reassurance and interaction may represent a key contributor to comfort, and partial disengagement through immersive AR may reduce this effect.

This hypothesis may also help explain the slightly higher retrospectively reported anxiety the day after procedure in the AR-group. Other factors may also play a role, including altered sensory perception during AR use, unfamiliarity with immersive environments, or differences in how procedural experiences are processed and recalled. In addition, the possibility of a type I error cannot be excluded. Importantly, absolute anxiety levels remained low, indicating that this finding is not of clinical concern but may reflect subtle differences in how procedural experience is processed and recalled.

These findings suggest that AR may be beneficial in selected patients but does not provide a consistent advantage in a well-tolerated awake ablation workflow.

### Clinical implications and future perspectives

The present results reinforce the concept of awake PFA as a viable and patient-centered approach. The combination of effective analgesia, preserved communication, and high patient acceptance supports broader implementation of conscious sedation strategies in suitable patients.

While AR may contribute to a modest reduction in opioid use, its role appears to be most relevant in selected patients rather than as a universal adjunct in awake ablation workflows. Instead, emphasis should be placed on structured communication, team experience, and individualized sedation titration.

To further validate these findings, a prospective randomized study comparing conscious and deep sedation strategies is planned. Such studies will be essential to define the optimal sedation approach and to assess generalizability across different centers and patient populations.

### Limitations

This study has several limitations. First, it was a single-center, non-randomized observational study, which may limit generalizability and introduce selection bias. Although patients were consecutively enrolled, sequential allocation to the control and AR-groups may have introduced temporal bias, including effects of a procedural learning curve and improved team–patient interaction over time.

Second, the sample size was moderate and sufficient to assess feasibility and patient-reported outcomes but may be underpowered to detect smaller differences or rare adverse events.

Third, patient-reported outcomes were assessed using numeric rating scales, which may not capture all dimensions of patient experience. Retrospective assessments at 1 h and 1 day may be influenced by recall bias, and no formal assessment of memory was performed.

Fourth, midazolam may have influenced retrospective perception of procedural discomfort due to its amnestic properties, although all patients remained responsive.

Fifth, prior patient familiarity with immersive technologies was not assessed and may have influenced perception and tolerance.

Sixth, only a single AR device was used in this study; therefore, the findings may not be generalizable to other AR systems, which may differ in immersion, usability, or patient interaction and could potentially influence patient experience.

Seventh, procedural differences between groups, including longer procedure duration and left atrial dwell time in the AR-group, may have influenced sedation requirements and patient-reported outcomes. In addition, the higher rate of posterior wall ablation may have contributed to differences in procedural burden. Both differences may partly reflect parallel protocol-driven workflows associated with the VOLT CE Mark Extended study, including mandatory waiting periods as well as a higher rate of posterior wall ablation, thereby introducing potential confounding when interpreting the effect of AR.

## Conclusion

Conscious sedation for AF ablation using the BiB-PFA system is feasible, safe, and associated with high patient satisfaction and recommendation without the need for escalation to deep sedation. Intraprocedural pain and anxiety were low, supporting awake ablation as a patient-centered approach.

The addition of augmented reality support was associated with modest reduced opioid requirements and improved perception of the procedural environment but did not further improve an already high level of overall patient experience. While AR may be beneficial in selected patients, its routine use does not appear necessary in this setting.

## Declaration of generative AI and AI-assisted technologies in the writing process

During the preparation of this work authors used ChatGPT in order to improve language. After using this tool/service, the authors reviewed and edited the content as needed and take full responsibility for the content of the publication. 

## Data Availability

The datasets generated and analyzed during the current study are not publicly available.

## References

[1] Chun K-RJ, Miklavčič D, Vlachos K, et al. State-of-the-art pulsed field ablation for cardiac arrhythmias: ongoing evolution and future perspective. Europace. 2024;26:euae134. 10.1093/europace/euae134 PMC11160504 38848447

[2] Tilz RR, Chierchia GB, Gunawardene M, et al. Safety and effectiveness of the first balloon-in-basket pulsed field ablation system for the treatment of atrial fibrillation: VOLT CE Mark Study 6-month results. Europace. 2025;27:euaf072. 10.1093/europace/euaf072 PMC12036658 40163671

[3] Lo M, Gambhir A, Sundaram S, et al. Safety and effectiveness of a novel balloon-in-basket pulsed-field ablation catheter for the treatment of paroxysmal and persistent AF: Volt-AF IDE trial acute results. Heart Rhythm. 2025;22:2524–33. 10.1016/j.hrthm.2025.04.037 40294733

[4] Wenzel J-P, Hatahet S, Abdessadok R et al. Differential inflammatory and myocardial biomarker response after pulsed field ablation for atrial fibrillation using balloon-in-basket versus pentaspline catheter. Heart Rhythm O2 2025:S2666501825004477. 10.1016/j.hroo.2025.09.003 PMC12800810 41541730

[5] Wenzel J-P, Abdessadok R, Hatahet S, et al. Balloon vs. balloon—comparison of hemolysis and renal markers after cryoballoon vs. ballon-in-basket pulsed field pulmonary vein isolation. Front Cardiovasc Med. 2025;12:1681098. 10.3389/fcvm.2025.1681098 PMC12479498 41035702

[6] Wenzel J-P, Abdessadok R, Eitel C et al. Catheter design matters: Hemolysis and renal function after pulsed field ablation with balloon-in-basket vs. pentaspline systems. J Interv Card electrophysiol [Internet] 2025 [cited 2025 Oct 10];. Available from: https://link.springer.com/10.1007/s10840-025-02152-2 10.1007/s10840-025-02152-2 PMC13645903 41066058

[7] Wenzel J-P, Hatahet S, Eitel C et al. Inflammatory and myocardial biomarker response following pulmonary vein isolation: Cryoballoon versus balloon-in-basket pulsed field ablation. J Cardiovasc Electrophysiol 2025. 10.1111/jce.70221 PMC12891760 41361918

[8] Tilz RR, Wenzel J-P, Eitel C et al. Awake and ablated: First experience with a balloon-in-basket PFA system without propofol. Heart Rhythm O2 2025:S2666501825005574. 10.1016/j.hroo.2025.11.011 PMC12925817 41737941

[9] Hitching R, Hoffman HG, Garcia-Palacios A, et al. The emerging role of virtual reality as an adjunct to procedural sedation and anesthesia: A Narrative Review. JCM. 2023;12:843. 10.3390/jcm12030843 PMC9917582 36769490

[10] Zako J, Daccache N, Morisson L, Richebé P, Laferrière-Langlois P. Virtual reality for reduction of intraprocedural pharmacological sedation and analgesia in adult patients: A systematic review and meta-analysis. Anaesth Crit Care Pain Med. 2025;44:101483. 10.1016/j.accpm.2025.101483 39862969

[11] Bruno RR, Lin Y, Wolff G, et al. Virtual reality-assisted conscious sedation during transcatheter aortic valve implantation: a randomised pilot study. EuroIntervention. 2020;16:e1014–20. 10.4244/EIJ-D-20-00269 32597390

[12] World Medical Association. World medical association declaration of Helsinki: Ethical principles for medical research involving human participants. JAMA. 2025;333:71–4. 10.1001/jama.2024.21972 39425955

[13] Tilz RR, Ukita K, Eitel C, Kuck K-H, Hatahet S, Wenzel J-P. How to isolate the superior vena cava using balloon-in-basket pulsed field ablation system: Standardized workflow and initial results. Heart Rhythm O2 2026:S2666501826000267. 10.1016/j.hroo.2026.01.014 PMC13107035 42038685

[14] Tilz RR, Hatahet S, Eitel C et al. Pulmonary vein isolation using a novel balloon-in-basket pulsed field ablation system: The Lübeck how-to protocol. Heart Rhythm O2 2026:S266650182600098X. 10.1016/j.hroo.2026.02.022 PMC13307493 42369783

[15] Hatahet S, Popescu S, Eitel C, et al. Biomarker response to balloon-in-basket pulsed field ablation: does posterior wall isolation matter? Front Cardiovasc Med. 2026;13:1733642. 10.3389/fcvm.2026.1733642 PMC12992039 41852605

[16] Tilz RR, Busch S, Chun KRJ, et al. Analgosedierung in der Kardiologie: Konsensuspapier der DGK und DGAI 2024. Kardiologie. 2024;18:187–99.

[17] Clivio S, Putzu A, Tramèr MR. Intravenous lidocaine for the prevention of cough: Systematic review and meta-analysis of randomized controlled trials. Anesth Analg. 2019;129:1249–55. 10.1213/ANE.0000000000003699 30169416

[18] Weibel S, Jokinen J, Pace NL, et al. Efficacy and safety of intravenous lidocaine for postoperative analgesia and recovery after surgery: a systematic review with trial sequential analysis. Br J Anaesth. 2016;116:770–83. 10.1093/bja/aew101 27199310

[19] Gajniak D, Mendrala K, Cyzowski T, Polak M, Gierek D, Krzych ŁJ. Efficacy of lidocaine infusion in high-risk vascular surgery—a randomized, double-blind, placebo-controlled single-center clinical trial. JCM. 2023;12:2312. 10.3390/jcm12062312 PMC10053864 36983312

[20] Ferreira-Valente MA, Pais-Ribeiro JL, Jensen MP. Validity of four pain intensity rating scales. Pain. 2011;152:2399–404. 10.1016/j.pain.2011.07.005 21856077

[21] Van Gelder IC, Rienstra M, Bunting KV, et al. 2024 ESC Guidelines for the management of atrial fibrillation developed in collaboration with the European Association for Cardio-Thoracic Surgery (EACTS). Eur Heart J. 2024;45:3314–414. 10.1093/eurheartj/ehae176 39210723

[22] Ekanem E, Neuzil P, Reichlin T, et al. Safety of pulsed field ablation in more than 17,000 patients with atrial fibrillation in the MANIFEST-17K study. Nat Med. 2024;30:2020–9. 10.1038/s41591-024-03114-3 PMC11271404 38977913

[23] Garcia R, Waldmann V, Vanduynhoven P, et al. Worldwide sedation strategies for atrial fibrillation ablation: current status and evolution over the last decade. Europace. 2021;23:2039–45. 10.1093/europace/euab154 34308973

[24] Chun KRJ, Plank K, Neven K et al. Characterization of sedation strategies in real-world use of pulsed field ablation Sub-analysis of the EU-PORIA registry. Europace 2025:euaf287. 10.1093/europace/euaf287 41248987

[25] Calvert P, Mills MT, Murray B et al. Feasibility of pulsed field ablation for atrial fibrillation under mild conscious sedation. J Interv Card Electrophysiol 2024. 10.1007/s10840-024-01961-1 PMC12436502 39623098

[26] Bolhuis RE, Bax IN, Van Dijk VF et al. Pulsed field ablation for atrial fibrillation with a balloon-in-basket system under conscious sedation: A retrospective feasibility and safety analysis. Heart Rhythm 2026:S1547527126001293. 10.1016/j.hrthm.2026.02.011 41713759

